# Firm Size and Employment during the Pandemic

**DOI:** 10.1177/2378023121992601

**Published:** 2021-02-12

**Authors:** Ken-Hou Lin, Carolina Aragão, Guillermo Dominguez

**Affiliations:** 1 The University of Texas, Austin, TX, USA

**Keywords:** firm size, employment, COVID-19, remotability

## Abstract

Previous studies have established that firm size is associated with a wage premium, but the wage premium has declined in recent decades. The authors examine the risk for unemployment by firm size during the initial outbreak of coronavirus disease 2019 in the United States. Using both yearly and state-month variation, the authors find greater excess unemployment among workers in small enterprises than among those in larger firms. The gaps cannot be entirely attributed to the sorting of workers or to industrial context. The firm size advantage is most pronounced in sectors with high remotability but reverses in the sectors most affected by the pandemic. Overall, these findings suggest that firm size is linked to greater job security and that the pandemic may have accelerated prior trends regarding product and labor market concentration. They also point out that the initial policy responses did not provide sufficient protection for workers in small and medium-sized businesses.

Half of American workers are employed in small and medium-sized businesses. A recent estimate indicates that nearly 30 percent of private sector workers are employed by firms with fewer than 50 employees and another quarter by businesses with 50 to 499 employees (Oslund 2019). Using establishment-level time-series data, a recent study also revealed a negative correlation between size and net job creation (Neumark, Wall, and Zhang 2011), indicating that small businesses remain important job creators in the U.S. economy.

Compared with workers in larger enterprises, workers in small and medium-sized businesses are more likely to be Hispanic, have low educational attainment, and live in rural areas (Headd 2000, 2010). Besides differences in characteristics, several studies have suggested that being employed by smaller enterprises is itself a source of disadvantage: similar workers tend to earn lower wages in smaller firms (Brown, Hamilton, and Medoff 1990; Hollister 2004; Villemez and Bridges 1988). The outbreak of coronavirus disease 2019 (COVID-19) in the second quarter of 2020 produced an unprecedented demand shock upon the U.S. economy. Much of the job loss concentrated in industries that have low remotability and were not recognized as essential (Papanikolaou and Schmidt 2020). Small businesses were particularly affected, as they are more likely to be in the service sector and have limited access to financial reliefs.

In this study we examine the connection between firm size and employment security during the pandemic. We find that the surge of unemployment between March and June 2020 was larger among workers in small firms than among those in large enterprises. The difference in excess unemployment cannot be fully attributed to the composition of workers or to industrial context. However, the association between firm size and job security is not constant. The firm size advantage was greater in industries with high remotability, but the pattern reversed in industries most affected by the outbreak, such that the smallest businesses retained their workers at slightly higher rates than larger firms.

Overall, this study indicates that firm size is positively associated not only with higher wages, as shown in prior studies, but also with greater employment security. The uneven impacts of the pandemic are likely to accelerate the market concentration in the United States (Autor et al. 2020; Philippon 2019; Sokolova and Sorensen 2021) and slow down the job recovery, particularly in industries with high remotability. It also shows that the initial policies developed to maintain the payroll of small businesses were insufficient in closing the employment gap between small and large enterprises. We conclude with the implications of these trends.

## Between-Firm Inequality

Organizations play an important role in determining the distribution of economic resources (Baron and Bielby 1980; Cobb 2016; Tomaskovic-Devey and Avent-Holt 2019). A large number of sociological studies have documented that workers in smaller enterprises earn lower wages, have worse working conditions, and receive less job benefit than those in larger firms (e.g., Kalleberg and Buren 1996; Lin, Bondurant, and Messamore 2018; Villemez and Bridges 1988). Although some of the disparities could be attributed to the sorting of workers into different firms, sizable inequalities persist even when comparing otherwise similar individuals.

Recent studies indicate that the firm size wage premium has declined substantially since the 1980s (Bloom et al. 2018; Hollister 2004). The decline is most salient among low-skilled workers, who used to receive a higher premium for working at larger firms (Cobb and Lin 2017). The decline is driven in part by the changing compensation practices among larger firms, moving from an internal labor market model to an external, market-wage scheme (Dencker and Fang 2016; DiPrete, Goux, and Maurin 2002).

The transformation occurred at a time of increased product and labor market concentration in the U.S. economy. The level of concentration has increased in multiple industries, including finance, retail, transportation, and telecommunication (Avent-Holt 2012; Bajgar et al. 2019; Crowley and Stainback 2019; Philippon 2019; Tomaskovic-Devey and Lin 2011). Several studies have pointed out the emergence of “superstar firms” across different sectors, marked by high product market share among a small number of firms. The rising market power is associated with declining labor’s share of income (Autor et al. 2020), inflated prices for consumers (Philippon 2019), and lower wages for suppliers (Wilmers 2018). The increased concentration could be due to scale-biased technological change—advancement that favors larger operation—as well as institutional barriers to the entry of new competitors (Calcagno and Sobel 2014; Gutiérrez and Philippon 2019).

In the meantime, other studies have shown a clear monopsony power of firms in the labor market: workers have few options when deciding where to seek employment (Sokolova and Sorensen 2020). The level of concentration is particularly high in rural areas (Azar et al. 2020), and there has been a clear upward trend of concentration in manufacturing (Benmelech, Bergman, and Kim 2018). Together, these findings suggest not only that large firms earn greater profits through their high market share but also that the weakened competition for workers discourages these firms from paying higher wages.

Although the firm size wage premium has declined over time, little is known regarding whether employment security varies across firm sizes. As large firms abandon the internal labor market model in pursuit of flexibility, one may expect a convergence of employment security across different types of enterprises (Bidwell 2013; Lin 2016). In the meantime, the rising concentration of product market may grant higher employment security to workers in larger firms than their counterparts in small businesses. The favorable conditions could also encourage large firms to retain more of their employees during economic downturns, as they represent opportunities to claim greater market share.

## COVID-19 and the Labor Market

The outbreak of COVID-19 in the second quarter of 2020 introduced an unprecedented demand shock to the U.S. labor market. The official unemployment rate (U-3) jumped from 4.4 percent in March 2020 to nearly 15 percent in April. Black households are more likely than White households to have members working in the health sector, while Hispanics tend to reside with individuals who are unable to work from home. The two populations were therefore more exposed to the pandemic (Selden and Berdahl 2020). Although unemployment rose more sharply among men in previous recessions, women were more affected in 2020 because of both a collapse of retail and service sectors and an increase in demand for care work in household (Alon et al. 2020).

In response to the economic impacts of COVID-19, the Coronavirus Aid, Relief, and Economic Security (CARES) Act and the Paycheck Protection Program and Health Care Enhancement Act (PPP) were passed in March and April, respectively, to soften the hardships faced by households and businesses. Separate programs were designed to assist small and large enterprises. A total of $669 billion was allotted to PPP to give out as forgivable loans to small businesses, sole proprietors, and independent contractors. The Main Street Lending Program allocated $500 billion to facilitate loans made to businesses with between 500 and 10,000 employees. Even larger enterprises with direct access to the credit market received various direct and indirect supports from the Federal Reserve.

Despite the enactment of these measures, the tiered system has been criticized for favoring large businesses over small ones from the onset (Judge 2020). Although larger firms enjoy multiple sources of relief, small businesses could seek support only from the PPP, which was underfunded in the initial CARES Act and depleted as early as April 15, 2020. As private banks originated PPP loans, the priority was given to more established businesses with connections to banks (Bartik et al. 2020a) and with outstanding loans (to prevent defaults and bank losses). Although whether the PPP was effective in preserving employment is still under debate (Chetty et al. 2020), it is clear that the most in need, smallest businesses were having difficulties accessing these loans. In cases in which smallest businesses applied for loans, they faced longer processing times and were less likely to receive approvals (Neilson, Humphries, and Ulyssea 2020). The concentration of the banking sector and the private intermediation of public services also led to inefficient distribution of credit across firms (Granja et al. 2020; Lin and Neely 2020).

**Figure 1.**
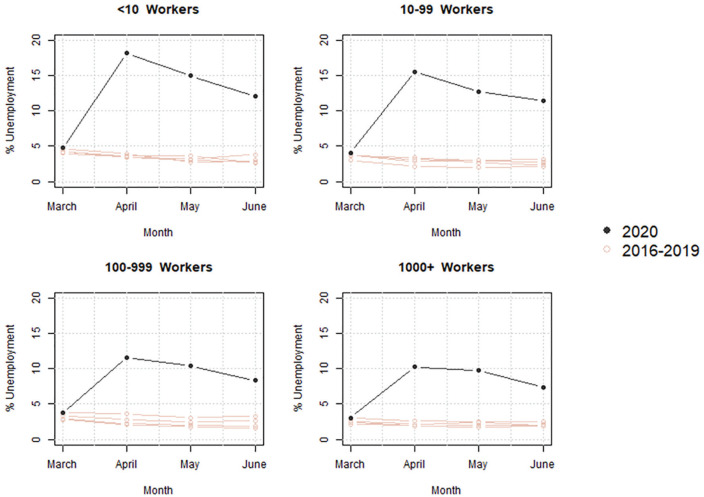
Comparing unemployment rates between 2020 and 2016–2019. *Note*: The estimates are generated from the Current Population Survey. The sample includes individuals aged 25 to 65 years who were employed in the private sector in the previous year, and firm size is derived from their prior employment. As individuals who were not previously employed are excluded from the sample, the aggregate unemployment rates are lower than the official, population rates for each month.

Figure 1 depicts the surge in unemployment between March and June by year and firm size among individuals who were employed in the preceding year (nonentrants). It shows that between 2016 and 2019, workers in large firms tend to have slightly lower rates of unemployment than those in small businesses. Unemployment began to surge dramatically in April 2020 compared with prior years. Yet the magnitude varies significantly by firm size. The unemployment among workers in firms with fewer than 10 employees increased most dramatically, exceeding 18 percent in April and remaining above 12 percent in June. In contrast, the unemployment rates among workers in firms with more than 1,000 employees increased to about 10 percent in April and declined to 7 percent in June.

The employment impact also varies across industrial sectors. The pandemic led to a sharp drop in demand for economic activities requiring face-to-face interaction. Although some activities were moved online, the ease of adjustment varies by the nature of the economic activities. Industries such as the finance and information sectors have less trouble transitioning online, whereas capital-intensive ones such as manufacturing, construction, and health are less equipped to move to remote work (Bartik et al. 2020b). Furthermore, some industries, such as agriculture and health care, were deemed critical to the proper functioning of the whole economy. These industries were often exempted from shutdown policies at either the state or municipal level. The exemption may reduce the impact on employment or signal the robustness of the demand of these sectors.

**Figure 2.**
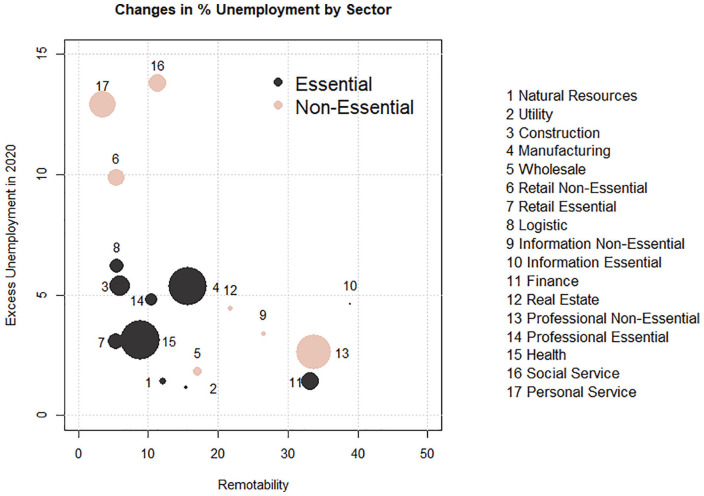
Unemployment rates by sector. *Note*: The estimates are generated by subtracting the average unemployment rates in March to June 2020 with the average of the same months in 2016 to 2019. Sizes indicate the number of workers. We categorize sectors as essential or nonessential on the basis of Papanikolaou and Schmidt (2020). Remotability is the weighted percentage of workers in the sector reporting that there are days they work exclusively from home in the American Time Use Survey 2017–2018 Leave Module.

Figure 2 contrasts the employment impact by sector (see Appendix A for a description of the industries). We calculate excess unemployment as the difference between the unemployment in March to June 2020 and the average in the same months between 2016 and 2019. Although the health crisis has led to a fast adoption of remote work arrangements, and the integration of work-home spaces (Schieman and Badawy 2020), the feasibility of remote work arrangements varies widely across economic sectors. We assign a score of remotability for each sector using the American Time Use Survey 2017–2018 Leave and Job Flexibilities Module. The scores represent the percentages of employees claiming that there are days they work exclusively from home. Each sector is also categorized as either essential or nonessential on the basis of the classification developed by Papanikolaou and Schmidt (2020). We split retail, information, and professional services into two separate sectors, as only parts of these sectors were deemed essential.

The figure shows that there was an increase of unemployment in 2020 for all sectors. It also indicates that the impact is moderated by both remotability and the essential status. The most affected sectors, such as social services, personal services, and retail nonessential, are those with low remotability. In the meantime, no high-remotability sectors experienced an increase of more than 5 percentage points. The importance of the essential status is clear when we compare the two retail sectors. Both having similar levels of remotability, the nonessential sector experienced an increase of 10 percentage points in unemployment, while the essential sector experienced an increase of unemployment of fewer than 4 percentage points.

These patterns suggest that the association between firm size and employment security could vary by sectorial contexts. Large firms in sectors with high remotability could capitalize on the existing technology and their scales, leading to higher retention of their workforce. Bartik et al. (2020b) found that 79 percent of firms with at least 100 employees had some workers transition to remote work, while this was the case for only 45 percent of businesses with fewer than 100 employees. In sectors with low remotability and declining demand, large firms may have more resources or credit access to withstand the harsh conditions. However, compared with smaller firms, large enterprises are also more able to redirect their resources to less affected divisions, leading to greater insecurity for workers performing face-to-face tasks.

## Empirical Analysis

### Data

We combine both the annual and monthly Current Population Survey (CPS) to examine how employment security varies by firm size (Flood et al. 2020).^1^ We merge the annual survey with the corresponding monthly surveys from March to June to link individuals’ prior and current employment status for 2016 to 2020. Depending on the rotation status of the individual, the annual survey could be matched to between one and four monthly records. Our primary sample includes individuals aged 25 to 65 years who were primarily private sector employees in the preceding year and assigned nonzero weight. We exclude public sector employees to avoid confounding firm size with sectoral differences regarding employment security. Our sample consists of a total of 391,915 individual-month observations that were in the labor force (either working or looking for a job).

We assess the impact of COVID-19 by comparing the prevalence of unemployment in March to June 2020 with the same months in the prior years, as well as exploiting the state-month variation in the spread of virus.^2^ We extract data from the COVID Tracking Project, which collects statistics directly from state and territory public health authorities.^3^ The data set has been widely used by studies in public health, medicine, and economics (e.g., Chetty et al. 2020; Kaashoek and Santillana 2020; Weinberger et al. 2020). The data set used in our analysis was downloaded on October 20, 2020.

### Variables

Our main dependent variable is an indicator of whether an individual is unemployed, defined as being in the labor force but not employed in a given month. When using this variable, the analysis includes only observations that were in the labor force. As many workers might be discouraged from seeking a job, we conduct a second set of analysis including those who dropped out of the labor force (*n* = 22,333) in Appendix B. The results are substantively similar.

Firm size is derived from the Annual Social and Economic Supplement, measuring the number of employees in a firm across all locations with seven categories. For simplicity, we collapse firm size into four categories: fewer than 10 employees (family or micro business), 10 to 99 employees (small), 100 to 999 employees (medium), and 1,000 or more employees (large). Although there may be meaningful variation among enterprises with more than 1,000 employers, we are unable to separate this category further because of top coding.

Table 1 describes the composition of workers by firm size. The patterns are consistent with previous findings (Headd 2000). Workers in smaller enterprises tend to receive lower wages, but there is significant variability among those in the smallest firms. They are also more likely to have lower levels of educations, with a higher proportion having only a high school diploma or less. Hispanics and men are more likely to work in smaller businesses than non-Hispanics and women. Overall, about 13 percent of our observations were employed in the smallest businesses, a quarter in firms with 10 to 99 workers, 20 percent in medium-sized firms, and 40 percent in firms with 1,000 or more workers.

**Table 1.** Composition of Workers by Firm Size.

	Firm Size			
	<10	10–99	100–999	≥1,000
Logged wage	2.811	2.954	3.120	3.198
*SD*	.782	.732	.734	.761
Age (years)	43.533	42.802	43.328	43.032
*SD*	11.499	11.553	11.447	11.544
Education				
Less than high school	.139	.103	.074	.049
High school	.316	.304	.268	.238
Some college	.273	.281	.271	.279
College	.192	.216	.253	.281
Advanced	.080	.096	.134	.154
Racial background				
Non-Hispanic white	.588	.606	.623	.625
Non-Hispanic black	.076	.099	.124	.131
Hispanic	.247	.213	.165	.144
Non-Hispanic Asian	.067	.058	.065	.079
Other	.022	.024	.024	.022
Men	.536	.552	.549	.525
Married	.586	.581	.607	.595
Parent	.496	.494	.500	.490
*n*	50,801	97,336	78,232	157,876

*Note*: Based on the observations in the labor force from 2016 to 2020.

To account for the positive selection of workers into larger firms, we include both educational attainment (five categories) and the average logged wage from the preceding year in the regression analysis. The latter is calculated as the total annual earnings by annual work hours. Both variables are expected to be negatively correlated with unemployment and joblessness. To address the sorting of workers on the basis of demographic and supply-side characteristics, we include racial background, age, gender, marital status, and parental status in our regression analysis. We also account for 8 occupational groups^4^ and 17 industrial categories (on the basis of the 2012 Census Classification Scheme and Papanikolaou and Schmidt 2020; see Figure 2 for the list and Appendix A for details) to avoid the confluence of firm size, occupation, and industry. It should be noted that the industrial categories describe the main activities at the respondent’s workplace, not the sector of the overarching firm.

Although past studies pointed out that union membership or contract could account for part of the firm size wage premium (e.g., Brown et al. 1990; Hollister 2004; Rosenfeld 2014), this measure is unfortunately not included in the Annual Social and Economic Supplement. As the level of unionization in the private sector has been low in recent years, we do not expect the omission would generate a large bias. Our sectorial analysis also indicates that the protective effect of firm size is particularly significant in industries with relatively low rates of union membership, suggesting that it is unlikely an explanation for the disparity between firms.

We use the total number of new positive COVID-19 cases per 1,000 residents to measure the severity of pandemic in the state-month. Positive cases include both confirmed and probable cases. The confirmed cases consist of people who receive positive results from polymerase chain reaction tests or other nucleic acid amplification tests. The probable cases consist of individuals who (1) have a presumptive laboratory test (i.e., antigen test), (2) have clinical and epidemiological evidence with no confirmatory testing, or (3) receive death certificates with COVID-19 as a cause of death with no confirmatory laboratory testing performed for COVID-19. We also conduct analysis with only either the confirmed cases or related deaths. The results are substantively similar.

**Figure 3.**
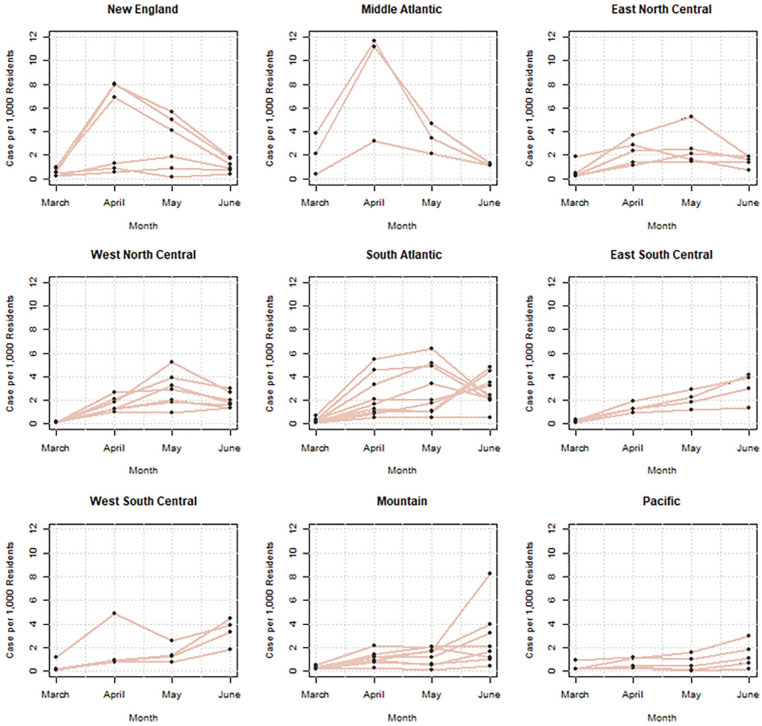
Positive cases per 1,000 residents by census division. *Note*: The estimated are based on the COVID Tracking Project. Positive cases include both confirmed and probable cases. The states and the District of Columbia are grouped on the basis of the census divisions.

Figure 3 describes the severity of the pandemic and regional variance. It shows that the spread was particularly serious in Middle Atlantic (New York and New Jersey) and New England states such as Connecticut, Massachusetts, and Rhode Island during the month of April. In these states, there were as many as 8 to 12 new positive cases per 1,000 residents in a month. Other states saw the outbreaks in later months, particularly Illinois in the East North Central, Nebraska in the West North Central, and Arizona in the Mountain states. These within-state variations allow us to assess the impact of COVID-19 on employment in 2020.

### Analytical Approach

We exploit both yearly and state-month variation to assess how the employment consequence of pandemic varies by firm size. As unemployment is a tail event, we estimate the effect of COVID-19 with a series of logistic regressions. Our year-to-year model, including observations from 2016 to 2020, is specified as

(1) $$\mathrm{Logit}\left(U\right)\;=\alpha +\beta_{1}F+\beta_{2}P+\beta_{3}FP+\beta_{i}X_{i}+\beta_{j}X_{i}P+\varepsilon ,$$

where *U* denotes the binary outcome of whether the worker is unemployed or jobless, *F* denotes three indicators for firm size, omitting the category of under 10 employees, *P* is an indicator for 2020, *FP* denotes the interaction terms between 2020 and firm size, *X_i_* denotes the controls described in the previous section, and *X_i_P* denotes their interaction terms. β_1_ captures the differences in employment security by firm size between 2016 and 2019, β_2_ captures the impact of the pandemic when other covariates equal to zero, and β_3_ captures the variation of impact by firm size. The logic is to compare the level of unemployment between 2020 and the previous years to gauge the impact of COVID-19, allowing the coefficients of the control variables to vary due to the pandemic.

Our month-to-month model, focusing only on the 2020 sample, is specified as

(2) $$\begin{array}{l} \mathrm{Logit}\left(U\right)\;=\alpha +\beta_{1}F+\beta_{2}C+\beta_{3}FC+\beta_{i}X_{i}\\ \;\;\;\;\;\;\;\;\;\;\;\;\;\;\;\;\;\;\;\;\;\;\;\;\;\;\;\;\;\;+\beta_{j}X_{i}C+\beta_{k}S_{k}\varepsilon , \end{array}$$

where *C* denotes the number of COVID-19 cases per 1,000 residents in the state-month, and *S_k_* denotes a series of indicators for the 50 states and the District of Columbia. β_1_ captures the differences in unemployment where there are no COVID-19 cases, β_2_ captures the per case effect when other covariates equal to zero, β_3_ captures the variation of effect by firm size, and β_*k*_ captures state fixed effects. Essentially, we compare how unemployment correlates with the spread of the virus by firm size within each state in 2020.

Because of the large number of interaction terms included in the models, the coefficients cannot be interpreted in the conventional fashion. To ease the comparison, we present the average marginal effects of the pandemic by firm size in the next sections (Breen, Karlson, and Holm 2018; Long and Mustillo 2018; Mood 2010). These effects are computed by contrasting the predicted probabilities by firm size and the severity of pandemic, while other population characteristics remain unchanged in all these scenarios. Coefficients (in logged odds) and associated standard errors are reported in Appendix C. In Appendix D, we perform a robustness check with a series of linear probability models (LPMs) with individual fixed effects. The results are consistent with the findings reported here.

## Findings

### The Impact of COVID-19 by Firm Size

**Figure 4.**
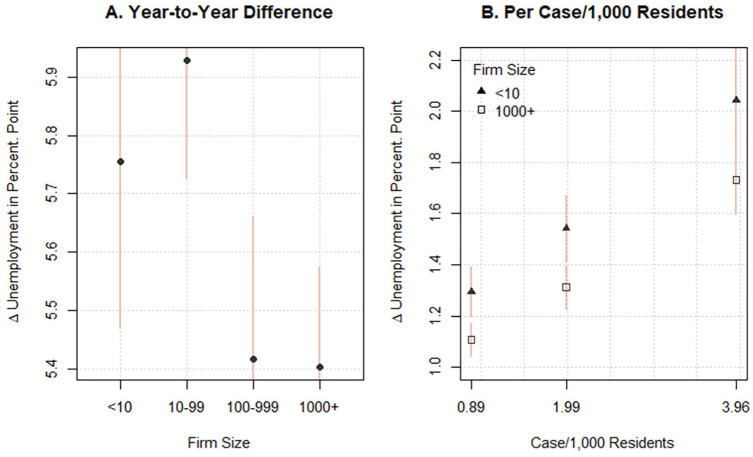
The unemployment impact of the pandemic by firm size. *Note*: The year-to-year estimates are based on equation 1, and the impact per case estimates are based on equation 2. All estimates are conditional on logged wage, age, education, racial background, occupation, sex, marital, and parental status. The average marginal effects are generated by computing and contrasting predicted probabilities with the population characteristics. Because of the logistic transformation, the absolute effect sizes differ when the covariates are set at different values.

Figure 4 presents the average marginal effects of COVID-19 by firm size. The year-to-year estimates compare 2020 with 2016 to 2019, while the per case estimates assess the marginal unemployment impact of an increase in positive case per 1,000 residents. In both accounts, we find that the impact of the pandemic is greater for micro and small businesses than medium and large companies. Figure 4A shows that in 2020, workers in the smallest enterprises experienced an increase of 5.7 percentage points in excess unemployment rate, with 2016 to 2019 serving as the baseline. In contrast, the excess unemployment among workers in firms with more than 1,000 employees is a half percentage point lower, about 5.4 percentage points.

Figure 4B presents the marginal changes in percentage points per case per 1,000 residents in three scenarios: when the spread is limited (25th percentile in our state-month sample = 0.89 cases/1,000 residents), when it is prevalent (median = 1.99 cases/1,000 residents), and when it is severe (75th percentile = 3.96 cases/1,000 residents). It shows that in all three scenarios, an increase of 1 case per 1,000 residents affects workers in the smallest enterprises more than workers in firms with at least 1,000 employees. The gap is largest in the severe scenario: for every 1 case increase per 1,000 residents, unemployment increases by more than 2 percentage points for workers in small businesses and by 1.7 percentage points for those in the largest enterprises. In Appendix B, we present the estimates for joblessness. The results are substantively similar.

Overall, this set of findings indicates that workers in smaller enterprises were more adversely affected by the COVID-19 pandemic, and the differences cannot be entirely attributed to their individual characteristics or industrial sectors.

### Sectorial Variation

As the impact of the pandemic is highly uneven across industries, our second set of analyses examines whether the pattern varies by sector. The analysis divides the sample into three major sectors on the basis of remotability and essential status (Figure 2). The high-remotability sector consists of all sectors with remotability scores greater than 20, including finance and nonessential professional services. The low-remotability and essential sector includes manufacturing, health, construction, essential retail, and four other sectors in the quadrant. The low-remotability and nonessential sector includes nonessential retail, social services, and personal services. This sector was also most affected by the pandemic during the months of interest.

**Figure 5.**
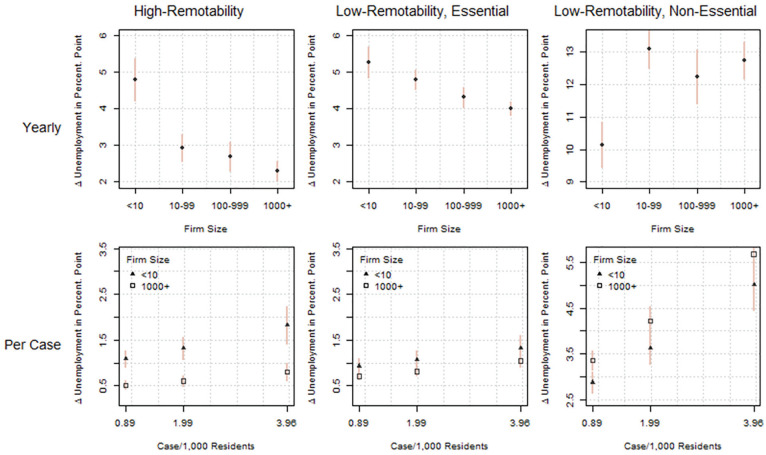
The unemployment impact of the pandemic by sector. *Note*: The year-to-year estimates are based on equation 1, and the impact per case estimates are based on equation 2. All estimates are conditional on logged wage, age, education, racial background, occupation, sex, marital, and parental status. The average marginal effects are generated by computing and contrasting predicted probabilities with the population characteristics. Because of the logistic transformation, the absolute effect sizes differ when the covariates are set at different values.

Figure 5 presents the estimates on the basis of both the yearly (top panel) and monthly (bottom panel) models. The top panel shows that the positive association between firm size and job security is largest among workers in the high-remotability sector. The excess unemployment for those employed in the smallest firms was about 5 percentage points. In contrast, the excess unemployment was about 3 percentage points lower among those employed by the largest firms, close to 2 percentage points. Yet the pattern reverses in the low-remotability and nonessential sector, such that workers in the smallest firms experienced the least excess unemployment compared with workers in larger firms. The excess unemployment in 2020 for the former is 10.1 percentage points, compared with 12.7 for the largest companies.

We see similar patterns in the bottom panel of Figure 5, in which the estimates are based on monthly variation. Again, the protective effect of firm size is most pronounced in the high-remotability sector. The marginal effects of a new case increase per 1,000 residents range from 1.1 to 1.8 percentage points among workers in the smallest firms, compared with an increase of 0.5 to 0.8 percentage points among workers in the largest firms. There is also the same reversal in the low-remotability, nonessential sector, such that the marginal effects of COVID-19 among the smallest firms are similar or smaller than those among the largest firms.

## Discussion

A growing literature has examined the uneven impacts of COVID-19 across the U.S. population (e.g., Alon et al. 2020; Bartik et al. 2020a, 2020b; Landivar et al. 2020; Papanikolaou and Schmidt 2020; Price-Haywood et al. 2020). In this study we assess the employment consequences by firm size during the initial outbreak. We find that unemployment increased more sharply among workers in firms with fewer employees, and the differences across firm sizes were not driven entirely by the composition of workers.

The association between firm size and employment security is most pronounced in industries with high remotability. In the most affected sectors, however, the smallest firms retained their workforce at higher rates than large enterprises. Overall, these findings show that workers in larger firms receive not only a wage premium (Cobb and Lin 2017; Hollister 2004) but also greater job security than their counterparts in small firms. Organizations continue to play a critical role in determining the economic well-being of workers during the pandemic (Tomaskovic-Devey and Avent-Holt 2019).

The finding that the smallest businesses retained more of their workers in the most vulnerable sectors is unexpected. One potential explanation is that larger firms may have greater flexibility to relocate the resources to less affected divisions. They could be more willing to let go of employees who normally perform face-to-face tasks. A second probable explanation is that the smallest enterprises are more likely to be organized around a family or individuals with close ties, which could reduce the dismissal of workers during economic downturns. In any case, the reversal is consistent with the finding that being employed by a large firm now provides less benefit to low-skilled workers (Cobb and Lin 2017), who tend to perform tasks with less remotability.

A recent study showed that many workers displaced by the pandemic have sought employment in the gig economy (Yildirmaz, Goldar, and Klein 2020) for financial relief. This suggests that our analysis underestimates the displacement impact of the pandemic, particularly for those with little savings. Although these workers would appear “employed” in the data, their work conditions are characterized by instability, precarity, and earning volatility (Benach et al. 2014; Schneider and Harknett 2019).

Scholars have called attention to the multiple pathways along which the pandemic has deepened existing inequalities (Alon et al. 2020; Coughlin et al. 2020; Davis, Hansen, and Seminario-Amez 2020; Gourinchas et al. 2020; Wrigley-Field 2020). Our results build on these findings by highlighting how the pandemic may have entrenched prior trends regarding market concentration (Azar et al. 2020; Gutiérrez and Philippon 2019), particularly in industries with high remotability. As more small businesses fail or cut back their workforces, one would expect an even more hostile environment when the pandemic is contained. An further increase in product and labor market concentration is likely to worsen consumer welfare, undermine community development, and reduce labor’s share of income (Autor et al. 2020; Crowley and Stainback 2019; Gereffi and Christian 2009), with also relevant implications for future job creation (Neumark et al. 2011).

Our results make clear that the policy response to the COVID-19 was insufficient to protect small and medium-sized businesses and their workers. To reverse these trends, further assistance needs to be provided to these businesses in the form of payroll-based grants, not loans. This will drastically reduce the uncertainties associated with the subsidy and the inability to target businesses truly in need of the funds.

## Figures and Tables

**Figure B1.**
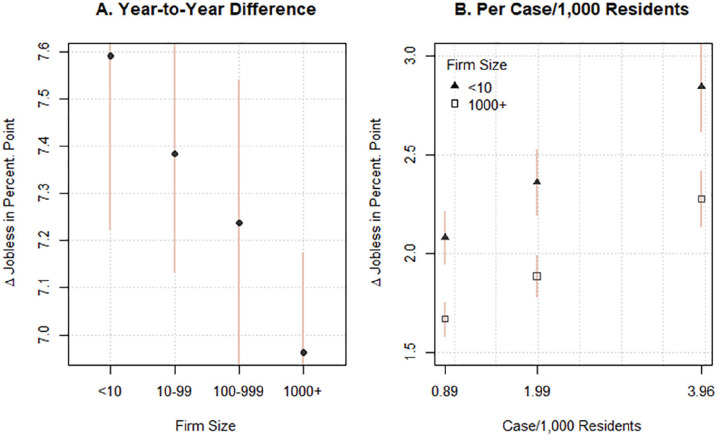
The impact of the pandemic on joblessness by firm size.

**Figure B2.**
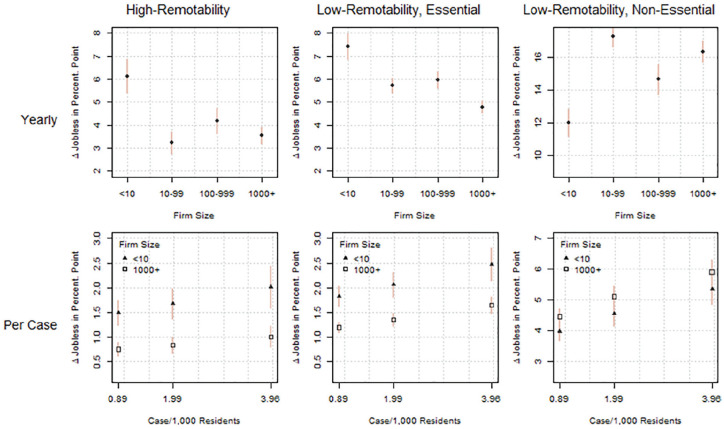
The impact of the pandemic on joblessness by sector.

**Figure D1.**
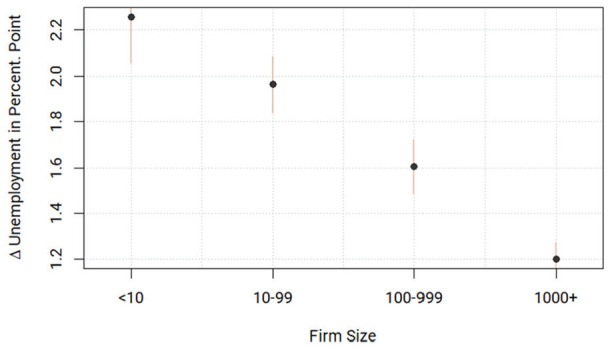
Unemployment impact by firm size with individual fixed effect.

## Appendix A

**Table:** Industrial Classification.

Industry Groups	Examples of Industries Included	Remotability	Essential Status
1. Natural resources	Crop and animal production; logging, fishing, hunting, and trapping; mining	Low	Yes
2. Utility	Electric power generation, transmission and distribution; water, steam, air conditioning, and irrigation systems; sewage treatment facilities	Low	Yes
3. Construction		Low	Yes
4. Manufacturing	Nondurable goods manufacturing (animal food, grain and oilseed milling; petroleum refining) and durable goods manufacturing (machinery manufacturing; electronic component and product manufacturing)	Low	Yes
5. Wholesale trade	Durable goods (motor vehicles, parts and supplies, merchant wholesalers; machinery, equipment, and supplies, merchant wholesalers) and nondurable goods (groceries and related products, merchant wholesalers; alcoholic beverages, merchant wholesalers)	Low	No
6. Retail noncritical	Department stores and discount stores; automobile dealers; furniture and home furnishings stores	Low	No
7. Retail critical	Pharmacies and drug stores; grocery stores; gasoline stations	Low	Yes
8. Logistic	Air transportation; bus service and urban transit; truck transportation; postal service	Low	Yes
9. Information nonessential	Publishing, libraries and archive; data processing, hosting, and related services	High	No
10. Information essential	Wired telecommunications carriers; other telecommunications services	High	Yes
11. Finance	Banking and related activities; securities, commodities, funds, trusts, and other financial investments; insurance carriers and related activities	High	Yes
12. Real estate and leasing	Real estate; automotive equipment rental and leasing	High	No
13. Professional nonessential	Architectural, engineering, and related services; travel arrangements and reservation services; computer systems design and related services	High	No
14. Professional essential	Legal services; waste management and remediation services; elementary and secondary schools	Low	Yes
15. Health	Offices of physicians; offices of dentists; hospitals; nursing care facilities	Low	Yes
16. Social services	Community food and housing, and emergency services; child day care services	Low	No
17. Personal services	Museums, art galleries, historical sites, and similar institutions; traveler accommodation; restaurants and other food services; beauty salons	Low	No

## Appendix B: Estimates Predicting Joblessness

Our main analysis focuses on the demand-side factors and excludes observations dropped out of the labor force. One may find the approach overly conservative, as many workers stopped looking for a job because of the lack of openings. To provide a more inclusive measure for the impact of the pandemic, the analysis here includes all observations and uses joblessness (both looking and not looking for employment) as the dependent variable.

Figure B1 presents the estimates corresponding to Figure 4. As we use a more inclusive measure of the impact, the effect sizes here are larger than what are shown in the main analysis. Both the yearly and monthly estimates show that there is a shaper increase in joblessness among workers in smaller firms than workers in larger firms. Figure B2 presents the estimates corresponding to Figure 5. Again, we see the positive effect of firm size is most pronounced in the sector with high remotability. In the most affected sector, workers in larger firms were more likely to become jobless than those in smaller firms.

## Appendix C: Model Estimates

This appendix provides the coefficients (in logged odds) and standard errors for all the results presented in the analysis. Table C1 presents the coefficients and standard errors from equations 1 and 2. For each set of estimates, we present the main coefficients (β_1_, β_2_, and β_*i*_) on the left-hand side and the interaction coefficients (β_3_ and β_*j*_) on the right-hand side. Table C2 presents the estimates for firm size by the three major sectors. Because of the large number of interaction terms included in these models, these coefficients cannot be interpreted in the conventional fashion. For example, β_2_ in equation 1 appears negative, even though we expect that COVID-19 would have a positive effect on unemployment. This is because the coefficient represents the impact of the pandemic when all other covariates are zero, including age and logged wage.

**Table C1.** Estimates Predicting Unemployment, Full Sample.

	Equation 1, Full Sample				Equation 2, 2020 Sample			
	Main		Interaction		Main		Interaction	
	Coefficient	*SE*	Coefficient	*SE*	Coefficient	*SE*	Coefficient	*SE*
2020	–.563**	(.192)						
Case/1,000 residents					.217**	(.072)		
Firm size								
<10	—	—	—	—	—	—	—	—
10–99	–.101***	(.020)	.087**	(.033)	–.069*	(.033)	.022*	(.010)
100–999	–.057*	(.022)	–.009	(.037)	–.071	(.037)	–.009	(.012)
≥1,000	–.165***	(.021)	.057	(.034)	–.097**	(.034)	–.014	(.011)
Logged wage	–.256***	(.010)	.060***	–.015	–.191***	(.015)	–.017***	(.005)
Age/10	–.304***	(.051)	.187*	(.082)	–.107	(.082)	–.011	(.026)
Age squared/1,000	.323***	(.058)	–.175	(.093)	.152	(.093)	.003	(.029)
Education								
Less than high school	—	—	—	—	—	—	—	—
High school	–.219***	(.023)	.127***	(.038)	–.054	(.040)	–.035**	(.013)
Some college	–.212***	(.024)	.117**	(.040)	–.077	(.042)	–.025	(.014)
College	–.319***	(.028)	.042	(.046)	–.220***	(.047)	–.044**	(.015)
Advanced	–.358***	(.036)	.027	(.058)	–.230***	(.058)	–.062***	(.017)
Racial background								
Non-Hispanic white	—	—	—	—	—	—	—	—
Non-Hispanic black	.473***	(.019)	–.326***	(.032)	.361***	(.034)	–.078***	(.011)
Hispanic	–.135***	(.019)	.162***	(.029)	.019	(.031)	.008	(.009)
Non-Hispanic Asian	–.120**	(.033)	.542***	(.046)	.355***	(.043)	.015	(.011)
Other	.331***	(.039)	–.217***	(.065)	.158*	(.067)	–.015	(.030)
Supply side								
Male	—	—	—	—	—	—	—	—
Female	.400*	(.016)	.116***	(.025)	.158***	(.024)	.003	(.008)
Single	—	—	—	—	—	—	—	—
Married	–.492***	(.015)	.273***	(.024)	–.225***	(.024)	.006	(.008)
Childless	—	—	—	—	—	—	—	—
Parent	.069***	(.015)	–.101***	(.024)	–.066**	(.024)	.021**	(.008)
Occupation fixed effects	Yes				Yes			
Industry fixed effects	Yes				Yes			
State fixed effects					Yes			
*n*	391,915				66,586			

* *p* < .05. ***p* < .01. ****p* < .001.

**Table C2.** Estimates Predicting Unemployment by Sector.

	Equation 1				Equation 2			
	Main		Interaction		Main		Interaction	
	Coefficient	*SE*	Coefficient	*SE*	Coefficient	*SE*	Coefficient	*SE*
High remotability								
2020	2.044***	(.423)						
Case/1,000 residents			.351**	(.130)				
Firm size								
<10	—	—	—	—	—	—	—	—
10–99	–.056	(.048)	–.289***	(.080)	–.349***	(.082)	–.021	(.024)
100–999	–.041	(.050)	–.346***	(.085)	–.275**	(.089)	–.058*	(.027)
≥1,000	–.114*	(.046)	–.395***	(.076)	–.382***	(.078)	–.062**	(.023)
*n*	90,516				15,892			
Low remotability, essential								
2020	–.076	(.252)						
Case/1,000 residents					.159*	(.081)		
Firm size								
<10	—	—	—	—	—	—	—	—
10–99	–.216***	(.028)	.063	(.047)	–.336***	(.046)	.073***	(.016)
100–999	–.239***	(.030)	.005	(.05)	–.346***	(.052)	.048**	(.017)
≥1,000	–.430***	(.028)	.07	(.047)	–.387***	(.047)	.005	(.016)
*n*	220,685				39,014			
Low remotability, nonessential								
2020	.364	(.328)						
Case/1,000 residents					.503***	(.112)		
Firm size								
<10	—	—	—	—	—	—	—	—
10–99	–.057	(.039)	.279***	(.058)	.333***	(.057)	–.036*	(.018)
100–999	–.025	(.046)	.190**	(.070)	.307***	(.068)	–.076***	(.021)
≥1,000	–.114**	(.038)	.296***	(.058)	.136*	(.058)	.016	(.018)
*n*	80,714				11,680			

* *p* < .05. ***p* < .01. ****p* < .001.

## Appendix D: Individual Fixed-Effect Estimates

To ensure that the firm size differences are not driven by the sorting of individuals, one reviewer suggested that we estimate a series of fixed-effect models by firm size. We do not incorporate them into our main models, because the dependent variable is binary (i.e., either employed or unemployed) and because the observation window is narrowed (i.e., at most four months). And above all, the firm size measure does not vary within individuals. Taking a fixed-effect approach with a logistic regression necessitates dropping all the individuals that did not change their employment status because of perfect prediction. For our sample, more than 85 percent of the observations would have to be dropped with this approach, which introduces an obvious selection problem that is based on the dependent variable.

To circumvent the problem, we estimate a set of LPMs by firm size with individual fixed effects for cases for which we have at least two monthly observations. Although the LPMs may not be appropriate for tail events such as unemployment, the test provides an additional robustness check of our main finding that firm size is positively associated with employment security during the pandemic.

Figure D1 presents the estimates from these models. It shows that for every one case increase per 1,000 residents, the unemployment rate increases by more than 2.2 percentage points for those who worked in firms with fewer than 10 workers, about 2 percentage points for those in firms with 10 to 99 employees, about 1.6 percentage points for those in firms with 100 to 999 workers, and about 1.2 percentage points for those worked in the largest enterprises. These results are consistent with what we have reported in the main analysis.

## Notes

1 The CPS is a monthly household survey conducted by the Bureau of Labor Statistics and the Census Bureau to provide the most updated trends about the U.S. labor market. The CPS sample consists of eight rotation groups, each representative of the U.S. population. A group is in the sample for four consecutive months, leaves the sample for the next eight months, and then returns for another four consecutive months before exiting the survey. Households interviewed in March also answer the Annual Social and Economic Supplement and report their labor market experiences in the preceding year. The pandemic has led to a significant decline in response rate, especially for households that just entered or returned to the sample. As in-person interviews became infeasible in March 2020, all interviews for the subsequent months were conducted exclusively by phone. The Census Bureau also closed two call centers because of the pandemic and operated with more limited capacity. Together, these factors are likely to generate nontrivial bias to the 2020 sample. If the nonresponse is correlated with unemployment, we are likely to underestimate the association between firm size and job insecurity.

2 We use state-level variation because the economic and public health responses to the pandemic have been largely organized at the state level and because we do not have more detailed geographical information for respondents residing in less densely populated areas in the CPS.

3 See https://covidtracking.com for a detailed description.

4 We thank one of the reviewers for this suggestion. We use broad occupational categories, including professionals, managers, clericals, sales, crafts, operatives, service workers, and laborers.
